# Maxillary Incisor Fragment Reattachment Protocols: Influence on Tooth Fracture Resistance and Strength of Bonding to Orthodontic Brackets

**DOI:** 10.3390/jcm14093220

**Published:** 2025-05-06

**Authors:** Moataz Elgezawi, Rasha Haridy, Khalid S. Almulhim, Moamen A. Abdalla, Ahmed A. Alsulaiman, Laila Al Dehailan, Rasha Alsheikh, Shahad Alotaibi, Deena Alghamdi, Ohud Almutairi, Sahar F. Alwehaibi, Ala’a Kamal, Dalia Kaisarly

**Affiliations:** 1 Department of Restorative Dental Sciences, College of Dentistry, Imam Abdulrahman Bin Faisal University, P.O. Box 1982, Dammam 31441, Saudi Arabia; malgizawi@iau.edu.sa (M.E.); ksalmulhim@iau.edu.sa (K.S.A.); ldehailan@iau.edu.sa (L.A.D.); ralsheikh@iau.edu.sa (R.A.); 2 Department of Clinical Dental Sciences, College of Dentistry, Princess Nourah bint Abdulrahman University, P.O. Box 84428, Riyadh 11671, Saudi Arabia; 3 Department of Substitutive Dental Sciences, College of Dentistry, Imam Abdulrahman Bin Faisal University, P.O. Box 1982, Dammam 31441, Saudi Arabia; maabdalla@iau.edu.sa; 4 Department of Preventive Dental Sciences, College of Dentistry, Imam Abdulrahman Bin Faisal University, P.O. Box 1982, Dammam 31441, Saudi Arabia; abalsulaiman@iau.edu.sa; 5 College of Dentistry, Imam Abdulrahman Bin Faisal University, P.O. Box 1982, Dammam 3441, Saudi Arabia; 2180006378@iau.edu.sa (S.A.); 2180002439@iau.edu.sa (D.A.); 6 Department of Restorative Dentistry, Ministry of Health, Riyadh 12382, Saudi Arabia; ohud.mohammed@hotmail.com (O.A.); sahar.alwehaibi@gmail.com (S.F.A.); 7 Department of Restorative and Prosthetic Dentistry, Dar Al Uloom University, P.O. Box 45142, Riyadh 13314, Saudi Arabia; a.kamal@dau.edu.sa; 8 Department of Restorative Dentistry and Periodontology, LMU University Hospital, LMU Munich, 80336 Munich, Germany; dalia.kaisarly@med.uni-muenchen.de

**Keywords:** dental trauma, adhesive dentistry, incisal fragment reattachment, Ribbond band, fracture resistance, shear bond strength, flowable composite, composite buildup

## Abstract

**Objectives**: Trauma to maxillary incisors is frequent, and requires timely, conservative management for optimal prognosis. This in vitro study evaluated the fracture resistance (FR) and orthodontic bracket bond strength (BS) of incisors following incisal fragment reattachment using various restorative techniques. **Materials and Methods**: Two independent tests—FR testing (Newtons) and BS testing (megapascals)—were conducted. Eighty intact human maxillary central incisors (n = 40/test), standardized in size and shape using a digital caliper (Mitutoyo, ±0.01 mm), were embedded in acrylic resin and numbered. An uncomplicated crown fracture was induced in 64 teeth (n = 32/test), and the teeth were randomly assigned (simple randomization using Excel’s RAND function) to five groups (n = 8/group/test): (1) intact teeth (negative control, NC); (2) nanohybrid composite buildup using Filtek Z250 and Single Bond 2 (positive control, CB); (3) fragment reattachment using flowable composite (Filtek Supreme, FL); (4) reattachment with a palatal veneer using a nanohybrid composite (PV); and (5) reattachment reinforced with a polyethylene fiber band (Ribbond Inc., RB). In BS testing groups, stainless steel orthodontic brackets (PINNACLE) were bonded using Transbond XT, centered over the fracture line. Light curing was performed using an LED unit (Mini LED Standard, Acteon, 1250 mW/cm^2^, 20 s/bond, 40 s/composite, 2 mm curing tip distance). Specimens were stored in distilled water at room temperature for 24 h before reattachment. FR and BS were evaluated using a universal testing machine (Instron) until failure. Failure modes were analyzed, and data were statistically evaluated using one-way ANOVA, Tukey’s post hoc test, and Pearson’s correlation analysis. **Results**: Significant differences were observed among groups for both FR and BS (*p* < 0.05). The NC group exhibited the highest FR (514.4 N) and BS (17.6 MPa). The RB group recorded the second-highest FR (324.6), followed by the PV (234.6), CB (224.9), and FL (203.7) groups. The CB group demonstrated the second-best BS (16.6), followed by the RB (15.2), FL (13.4), and PV (6.5) groups. FR and BS were negatively correlated. Mixed failures predominated in the reattachment groups, except for the PV group, which showed mainly adhesive failures. In BS testing, mixed failures dominated in the NC and CB groups, while adhesive failures predominated in the PV and FL groups. **Conclusions**: Ribbond reinforcement improves the mechanical performance of reattached incisal fragments, and composite buildup may provide more reliable bracket bonding than fragment reattachment. **Clinical Relevance**: In cases where biomimetic, minimally invasive reattachment is indicated, Ribbond fiber reinforcement appears to offer a reliable restorative solution.

## 1. Introduction

Traumatic teeth injuries are not uncommon, especially in children and youths. The incidence of coronal tooth fracture has been estimated to constitute 92% of all permanent traumatic teeth injuries [1,2,3]. Of these, 80% and 16% affect the maxillary central and lateral incisors, respectively. The adverse consequences of traumatic injuries to teeth include functional and esthetic degradation, in addition to psychological distress. The main objective of restorative treatment plans is the conservative restoration of fractured teeth into biomechanically and esthetically integral units of the dental arch with optimum clinical effectiveness and durability [4]. The World Health Organization has classified dental fractures as uncomplicated fractures, which involve enamel and dentin, and complicated fractures, which include pulp exposure and/or periodontal involvement. Uncomplicated fractures are generally more frequent and require less extensive treatment [1,5]. In traumatized maxillary incisors with crown fractures, uncomplicated fractures involving only enamel and dentin are more common than complicated fractures involving enamel, dentin, and pulp [6,7].

When managing traumatic teeth injuries, many decision-making factors contribute to identifying the most suitable treatment for a particular case. These include the extent of fracture, especially for subgingival extensions and violation of the biological width; retaining a sound coronal tooth structure in both the vertical and horizontal directions; pulp involvement; the presence of a root and/or alveolar bone fracture; the type of occlusion; the availability of the fractured fragments and their storage medium; the presence of associated soft tissue injuries; and the socioeconomic status of the patient [1,3,8,9]. Treatment options for coronal incisor fractures include direct bonding with resin composites, with or without pins; veneers; or crowns and orthodontic bands [4].

A method for reattaching fractured fragments by employing the acid etching technique and using resin composite to reattach teeth fragments was reported by Tennery in 1978 [10]. The technique is conservative and allows for the following characteristics to be maintained: natural tooth translucency, opalescence, surface gloss, and other optical characteristics, such as the color stability of combined enamel and dentin, which can be challenging to mimic with synthetic tooth-colored restorative materials [11]. This is particularly true when considering the thickness and anatomical variations in the two optically different structures, enamel and dentin [11]. Usually, the fracture strength of the reattached incisal fragment is used to assess the best reattachment method. However, the literature shows increasing controversy regarding the best technique for the reattachment of fractured fragments [12]. Numerous methods can improve the fracture resistance of reattached fractured fragments and improve clinical reliability, including beveling, the creation of chamfers or grooves, or the use of a circumferential bevel to remove dentin from the detached fragment [10]. When direct bonding and circumferential chamfering were tested using different adhesives and intermediate materials, no single material or technique could match the impact strength of sound teeth. However, the combination of single bonding with a circumferential chamfer proved to be the best reattachment method [13].

Various studies advocate for maintaining the fragment’s hydration and wettability before reattachment, and have suggested different storage media and immersion times. One study showed that one-hour hydration before reattachment improves the fracture resistance of reattached samples [14], and another found saline to be a better medium for fragment hydration than milk [15].

Only a few studies with limited evidence have shown the promise of using polyethylene fiber Ribbond bands in the reattachment and reinforcement of fractured incisal fragments [16].

Certain forms of malocclusion can increase the incidence of traumatic dental injuries by two to three times in cases with increased overbite [17]. The risk of dental trauma even increases with proclination of the maxillary incisors, or trauma may take place during orthodontic treatment, with consequential clinical challenges. Moreover, conditions like bruxism and deep bite can initiate anterior teeth trauma and adversely affect the success and long-term durability of restorations to traumatized anterior teeth [18,19]. Recently, methods for the orthodontic treatment of traumatic dental injuries, including trauma during orthodontic treatment, have been reported [17]. However, there is an apparent lack of consensus among orthodontists regarding the optimal orthodontic treatment of traumatized teeth [20], and there is little evidence on the efficacy of bonding orthodontic brackets to traumatized teeth with reattached fragments. The combination of incisal fragment reattachment and orthodontic bracket bonding is underexplored, although it is crucial for successful clinical outcomes in post-trauma orthodontic treatment.

This study was designed to investigate the effects of three different fragment reattachment techniques on the fracture resistance (FR) of maxillary central incisors and bond strength (BS) to orthodontic brackets, as well as to analyze the correlation between FR and BS. The null hypotheses were the following: (1) the reattachment technique has no effect on the fracture resistance of maxillary incisors and bond strength; and (2) there is no correlation between fracture resistance and bond strength.

## 2. Materials and Methods

### 2.1. Collection of Teeth

A total of 80 human maxillary central incisors, of similar dimensions, recently extracted for periodontal reasons from 20-to-40-year-old patients, were used in this study. A digital caliper (Mitutoyo 500-196-30, Kawasaki, Japan) was used to select teeth of similar size. For this, IRB approval was attained from the research ethics committee of Imam Abdulrahman Bin Faisal University (IRB-2023-02-159). Teeth were examined with dental loups with 4.5× magnification; only teeth free of caries, restorations, intrinsic discoloration, cracks, and developmental defects, and with fully formed roots, were selected for this study. The collected teeth were cleaned with ultrasonic scalers to remove any debris and remaining tissue, disinfected with 0.5% sodium hypochlorite for 15 min, and then immersed in distilled water at room temperature until further use.

### 2.2. Sample Size Calculation

The current study used power analysis to calculate the sample size. A similar previous study [21] was used for the sample size calculations, utilizing mean 1 = 239.91, mean 2 = 375.74, SD = 93.57, power 80%, and a 95% confidence interval [21,22]. With a total of 80 collected teeth, a minimum sample size of n = 7 was considered acceptable. Our study, like previous investigations, used a sample size of n = 8 [23,24,25].

### 2.3. Teeth Mounting

Two sets of five study groups (n = 8) were created, one set for fracture resistance (FR) testing and another set for orthodontic bracket bond strength (BS) testing. Periodontal simulation was performed in specimens assigned for FR testing as follows: roots were dipped in molten wax (DUO DIP, YETI Dentalprodukte GmbH, Engen, Germany) to a level 2 mm below the cementoenamel junction (CEJ) in an electric dipping wax machine (hotty, Renfert Gmbh, Hilzingen Germany), producing a 0.2–0.3 mm thick wax layer. Roots were embedded in self-curing acrylic resin (Unifast III, GC Corp., Tokyo, Japan) at 2 mm below the CEJ. Teeth were retrieved from the acrylic bases, and the wax layer was removed from the roots and acrylic base using a steam cleaner (Wasi-Steam 2, Wassermann Dental-Maschinen, Hamburg, Germany). Polyether Adhesive (3M, Deutschland GmbH, Neuss, Germany) was painted over the root and inside the acrylic resin socket, half of the socket of the acrylic base was filled with polyether impression material (Impregum L Duosoft Quick, 3M, GmbH, Neuss, Germany), teeth were inserted 2 mm below the CEJ [26], and excess impression material was removed with a scalpel. Specimens assigned for BS testing were mounted in self-curing acrylic resin up to a level of 2 mm below the CEJ, without periodontal simulation, to confirm a firm grip of teeth in the acrylic base during the shear loading of orthodontic brackets.

### 2.4. Study Groups

This study included five groups for both testing sets, the FR and BS testing sets: the (1) negative control (NC) group, consisting of sound, unprepared teeth; (2) the positive control (CB) group, where the incisal segment was restored using a composite buildup; (3) the flowable composite (FL) group, for which incisal fragment reattachment was performed using flowable composite; (4) the palatal veneer (PV) group, where a palatal composite veneer was used for fragment reattachment; and (5) the Ribbond (RB) group, for which a polyethylene fiber ribbon (Ribbond) was used for reinforcement during fragment reattachment. After mounting, teeth were numbered and randomly assigned into the study groups for both study tests, the FR and BS testing sets, using Excel’s RAND function for simple randomization. Each tooth was then immersed in distilled water in coded closed vials at room temperature.

**Figure 1:**
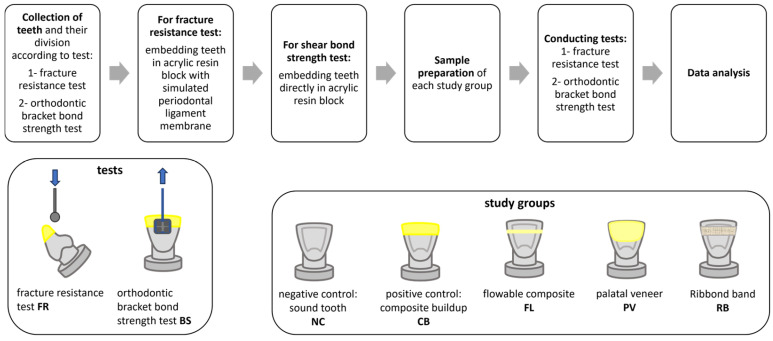
Workflow and study groups.

The workflow and the study groups are displayed in Figure 1.

### 2.5. Induced Incisal Fracture

In 64 teeth samples, an incisal fracture was induced, with 32 teeth per test group, the FR and BS testing groups, while 16 teeth were in the negative control group (n = 8/test). The silicone putty index (Express Putty Soft, 3M, Deutschland GmbH, Neuss, Germany) was measured for each tooth on the facial and proximal sides before the procedure to induce the incisal fracture, to serve as a guiding template during reattachment or composite buildup. The cutting line along the junction between the incisal and middle third was defined using a marker. An uncomplicated fracture of enamel and dentin was produced using a bi-sided porcelain diamond disk (22 mm in diameter, 0.2 mm thick, Diamantscheibe Superflex, Bredent Gmbh, Senden, Germany) perpendicular to the long axis of the tooth and parallel to the incisal edge [27]. The disk was mounted on a straight handpiece with water coolant flowing within a low-speed range. It was used in the labio-palatal direction to separate the incisal fragment at the predetermined level. Each specimen was stored with its fragment in distilled water at room temperature in the same coded vial for 24 h before the reattachment procedure [28].

### 2.6. Reattachment and Restoration Protocols

#### 2.6.1. Positive Control with Composite Buildup (CB)

Following the instructions of the manufacturer of the adhesive used [29], as well as previous reports [30], the fractured tooth surface was etched with orthophosphoric acid for 15 s, rinsed with water for 10 s, and gently air-dried for 5 s. Two coats of adhesive (Adper Single Bond 2 Adhesive) were applied, air-thinned, and light-cured for 20 s using an LED light curing unit (Mini LED Standard, Acteon, Mérignac, France) with a power output of 1250 mW/cm^2^. A nanohybrid composite (Filtek Z250, shade A2, 3M) was used to restore the fractured teeth to their original form. The putty index served as a guide for the composite buildup of the incisal segment, which proceeded as follows: First, a composite shell that fitted the treated tooth surface was built based on the index, and light curing was carried out for 40 s. Then, each increment underwent layering with light curing for 40 s until full tooth contour. Light curing was then carried out with the curing tip at a 2 mm distance. Finishing and polishing disks (Sof-Lex Extra Thin Contouring and Polishing Discs 12.7 mm, 3M ESPE, St. Paul, MN, USA) were used in a descending manner, from coarse to fine and ultrafine grits. The materials used in this study are listed in Table 1.

#### 2.6.2. Incisal Fragment Reattachment Using Flowable Composite (FL)

In each specimen, the opposing fracture surfaces of the separated incisal fragment and the tooth were etched, followed by adhesive application and light curing, as in CB, with the putty index serving as a reassembly guide. A layer of flowable composite (Filtek Supreme flowable composite, 3M) was applied to reattach the incisal fragment to the tooth, followed by light curing for 40 s, and then excess flowable composite was removed using a scalpel.

#### 2.6.3. Incisal Fragment Reattachment Using a Palatal Composite Veneer (PV)

The incisal fragment was initially reattached to its corresponding tooth, as follows: the fractured surfaces were first etched, followed by adhesive application and light curing, like with CB, with the putty index serving as a reassembly guide. The palatal surface was prepared to accommodate the resin composite veneer, and the preparation extended incisally to just before the incisal edge, gingivally to the junction between the middle and cervical third in the palatal fossa, and proximally to the mesial- and distal-line angles. For the preparation, a cut was made to a depth of 0.3 mm, using the half-depth of a round-shaped diamond bur (CD-50F, DIA-BURS, MANI, Tochigi, Japan). Then, for the complete preparation, a football diamond bur (FO-25, DIA-BURS, MANI, Japan) was used to a depth of 0.5 mm, as well as a chamfer finish line. The preparation was then etched, followed by adhesive application and light curing. A nanohybrid composite (Filtek Z250) was applied, contoured, and light-cured for 40 s. Finishing and polishing were performed as described for CB.

#### 2.6.4. Incisal Fragment Reattachment Using a Ribbond Band (RB)

Each tooth was reassembled by the reattaching the incisal fragment using the same etching and bonding procedure as used in the PV procedure. A palatal slot was prepared, with a depth of 1.2 mm, extending 3 mm in the inciso-gingival dimension, and located in a centralized manner to the reattachment line, extending proximally to the mesial- and distal-line angles of the tooth. The preparation started by establishing 2 mesiodistal depth grooves of 0.6 mm using the full depth of a round diamond bur (CD-50F, DIA-BURS, MANI, Japan), followed by carrying out the final stage of preparation using a wheel diamond bur (WR 13, DIA-BURS, MANI, Japan). Etching and adhesive application were carried out on the prepared slot as mentioned above. The mesiodistal width of each palatal slot was measured with a periodontal probe, and a piece of the same length was cut from the ribbon. The cut piece of ribbon was wetted with adhesive (Adper Single Bond 2 Adhesive). The nanohybrid composite Filtek Z250 was then applied in the palatal slot, the wetted ribbon was fitted and packed into the slot, and excess composite was removed, followed by light curing for 40 s. A final layer of nano-filled composite was applied to cover the ribbon, with further curing for 40 s. Finishing and polishing were performed as described for CB.

### 2.7. Fracture Resistance (FR) Test

Fracture resistance was tested using a universal testing machine (Instron, model 5965, Norwood, MA, USA) at a speed of 1.0 mm/min. Loading was applied to the palatal surface of the maxillary incisors, at a 45° angle to the tooth’s long axis, at the reattachment line, using a round-ended steel rod with a 5 mm diameter [31]. The fracture strength was recorded in Newtons [32], and the data were statistically analyzed.

### 2.8. Orthodontic Bracket Bond Strength (BS) Test

A stainless steel orthodontic bracket (PINNACLE™, Ortho Technology Inc, West Columbia, CA, USA) was bonded to the labial surface so that the reattachment line fitted in the center of the bracket base. The bracket attachment area was etched for 15 s and rinsed thoroughly. For CB, the composite buildup was roughened at the site of bracket bonding with a diamond stone before etching. An orthodontic primer (Transbond XT Light Cure Adhesive Primer, 3M Unitek, Monrovia, CA, USA) was applied and light-cured. Bracket adhesive cement was injected onto the base of the bracket, and then the bracket was placed over the etched surface and firmly seated, centralized over the reattachment line. Excess cement was removed with a dental explorer before light curing. The curing tip was applied 2–3 mm proximally to the bracket and curing was performed for 5 s each on the mesial and distal sides.

The shear bond strength of the orthodontic bracket was tested using the methodology applied by Abu Haimed et al. [33], with a universal testing machine (Instron, model 5965, USA). The bonded bracket was tied with ligature wire to a hook attached to a 500 N load cell. The test was performed at a static pull speed of 2 mm/min. The shear bond strength was calculated by dividing the force by the bonded bracket area (11.9 mm^2^). The bonded surface area (bracket base/central incisor) was calculated as the percentage area occupied by the bracket within the rectangular perimeter touching the bracket boundaries [34]. Image analysis software (ImageJ, Windows version bundled with 64-bit Java 8 software program, National Institutes of Health, Bethesda, MD, USA) was used to analyze and calculate the pixels and percentages, respectively, of images taken with a DSLR camera (EOS 250d, Canon, Tokyo, Japan), counting the pixels inside and outside the rectangle [28]. Mean bond strength values were recorded in megapascals (MPa), and standard deviations were calculated for each study group and statistically analyzed.

### 2.9. Failure Mode Analysis

The failure mode along the interfaces was inspected using a 4.5× magnifying dental loupe and classified into the following categories: (1) cohesive failure, indicating failure within the intermediate composite, composite buildup, or adhesive bond, or purely within the fragment; (2) adhesive failure, indicating failure at the interface between the fractured tooth and the intermediate composite, buildup composite, or resin adhesive; (3) mixed failure, indicating a combination of adhesive and cohesive failures occurring at different locations, with or without the involvement of the substrate tooth structure; and (4) failure within the substrate tooth, indicating pure structural failure of the tooth. In cases involving brackets, failure modes were categorized as follows: (1) cohesive failure; (2) adhesive failure; and (3) mixed failure, occurring either between the two reattached segments (with the bracket attached to the incisal segment), or between the bracket and the tooth.

### 2.10. Statistical Analysis

Data were analyzed using one-way ANOVA, with a post hoc Tukey’s test for multiple comparisons, using SPSS statistics for Windows, version 29 [8,28], at a level of significance of *p* < 0.05. Pearson’s correlation analysis was conducted to evaluate the relationship between tooth fracture resistance and orthodontic bond strength at a 0.05 level of significance (two-tailed).

## 3. Results

### 3.1. Fracture Resistance and Orthodontic Bracket Bond Strength

Table 2 summarizes the fracture resistance (FR) and shear bond strength (BS) results. The NC group showed the highest FR (514 ± 19.4 N), followed by the RB group (324.6 ± 20.9 N). No statistical significance was observed between the PV and CB groups, while the FL group showed the least fracture resistance (203.7 ± 7.8N). In terms of orthodontic bracket BS, the NC group had the greatest values (17.6 ± 0.6 MPa), followed by the CB (16.6 ± 0.5 MPa), RB, and FL groups, while the PV group had the lowest value (6.5 ± 0.9 MPa), with significant differences between all groups. Pearson’s correlation analysis indicated a negative correlation between FR and BS (r(df) = −0.868, *p* < 0.001).

### 3.2. Fracture Resistance Failure Mode

The failure modes of the FR group are listed in Table 3. The PV group had the highest percentage (62.5%) of adhesive failures, followed by the RB group, which showed an equal distribution of substrate and mixed failures (each at 37.5%) and adhesive failures (25%). No cohesive failures were observed. Substrate failure was more prominent in the RB group compared to the other groups. The CB group exhibited a mixed failure of 37.5%, characterized by the detachment of part of the composite buildup along with some of the tooth structure, while the remaining composite remained attached to the tooth. The FL group predominantly showed mixed failure (50%), with complete detachment of the incisal fragment and no substrate failure.

### 3.3. Bond Strength Failure Mode

Table 4 and Table 5 summarize the failure modes and the frequency of incisal fragment detachment during orthodontic bracket debonding, respectively. The NC (negative control) and CB (composite buildup) groups exhibited adhesive and mixed failures without any involvement of the tooth structure. In the NC, CB, and RB (Ribbond band) groups, mixed failures predominantly occurred, characterized by detachment of the bracket from the reattachment assembly without incisal fragment detachment. Notably, none of the tested groups demonstrated any cohesive failures.

In the FL (flowable composite) group, adhesive failure was the most prevalent mode, followed by mixed failure. Mixed failures in this group were characterized by detachment of the incisal fragment along with the bracket, while the bracket remained attached to the detached fragment. In the PV (palatal veneer) group, failures were predominantly adhesive or mixed, with an equal incidence of bracket detachment alone and detachment of the incisal fragment along with the bracket.

Table 5 further illustrates the frequency of incisal fragment detachment upon bracket debonding across the different groups. In the CB group, no cases of incisal fragment detachment were recorded, and all failures were limited to bracket debonding without fragment detachment. In contrast, the FL group showed a higher frequency of incisal fragment detachment during bracket debonding (six cases with fragment detachment vs. two cases without). The PV group exhibited an equal distribution between debonding with and without incisal fragment detachment (four cases each), and the RB group predominantly showed bracket debonding without incisal fragment detachment (seven cases without vs. one case with).

## 4. Discussion

Children and young adults are the principal populations affected by dental trauma to the maxillary anterior teeth, requiring esthetically, biomechanically, and functionally reliable restoration over long-term clinical service [35]. Uncomplicated crown fractures of the upper anterior teeth, which do not involve pulp exposure, are among the most common types of dental trauma, with enamel and dentin fractures accounting for 42.7% of all crown injuries [22].

Advancement in adhesion has enabled the development of conservative, minimal-intervention techniques for the reattachment of fractured fragments that aim to optimally restore the esthetic and biomechanical integrity of the traumatized tooth. The biomimetic approach, which involves mimicking the natural tooth structure as closely as possible, enables fractured fragments to be reattached effectively and durably [35].

Various techniques are recommended for reattaching uncomplicated fractured incisal fragments. These include the use of bonding agents only, an intermediate material (mainly flowable composite), a labial and/or palatal bevel [36], a circumferential chamfer [13], palatal veneering [37], and fiber-reinforced resin composite anchors [38]. As there is no general agreement on the best method for reattaching incisal fragments, the efficacy of the different reattachment techniques proposed needs to be investigated [27,35]. Comparison of the fracture resistance of reattached teeth and sound teeth has long been used as an indicator of the ability of reattached incisal fragments to withstand further trauma. Successful orthodontic treatment relies solely on the appropriate application of sustained forces on teeth using orthodontic brackets, to allow tooth movement via alveolar bone remodeling [39]. In this context, the effectiveness of orthodontic bracket bonding to restored traumatized teeth must be investigated [40]. The literature lacks information regarding the bond strength of orthodontic brackets to incisors with reattached incisal fragments. Our findings that the NC group of sound teeth exhibited significantly higher fracture resistance than all other test groups are in line with those of previous studies [13,41,42].

Ribbond is made of reinforced glass-fiber–polyethylene ribbon, and is used to reinforce temporary restorations, composite bridges, and resin restorations in extensive cavities [43]. Its long-term reinforcing potential [44] is indicated by improvements in fracture toughness and impact resistance [45,46]. Palatal Ribbond has been applied in a few case reports for the reattachment of fractured incisal fragments [47] of vital maxillary incisors. However, the literature lacks adequate reports on polyethylene fibers in reattaching uncomplicated fracture fragments of maxillary incisors [48]. Nevertheless, Sreen et al. studied the fracture loading of maxillary incisors after the reattachment of incisal fragments, and found an improved reinforcing effect when two fiber posts were placed in two vertical labial grooves, compared with using two vertical polyethylene fibers [49]. The results of the current study indicate that the RB group exhibited the highest FR among all the experimental groups. The low adhesive failure rate (25%) further highlights the unique load-bearing capacity of the complex when the Ribbond polyethylene fiber band was used.

Favorable stress distribution and stress transfer over a loaded complex with a greater surface area, and the ability of polyethylene fibers to cross-link structural discontinuity at the fragment reattachment site with stress relaxation potential [50], can explain these findings [51]. Moreover, the finely threaded, peculiarly cross-linked leno-woven ultra-high-molecular-weight threads could play a role in palatal anchorage and crack-inhibiting potential [52].

Jadhav et al. found that a single polyethylene fiber Ribbond reinforced the resin composite buildup of the fractured incisal edge of maxillary incisors when added to the palatal side of a resin composite with cohesive failure in the restored tooth [52]. Our findings that the palatal veneer group (PV) was the second-best-performing group in terms of FR are in general agreement with those of Zhang et al. The authors conducted a finite element analysis, and found that lingual chamfers, rather than labial chamfers, improved the strength of the adhesive joint in reattached incisors, and that the joint design mattered more than increasing the bonding area [53]. The interfacial polymerization contraction stresses of the palatal resin composite might have weakened the already-vulnerable adhesive interface, providing a probable explanation for the prevailing adhesive failure of the PV group in the FR test [54].

Moreover, the low performance in FR with the use of flowable composite (FL) as an intermediate resin, with the mixed mode of failure prevailing at 50%, points to a lower load-bearing capacity for the loaded complex. This may be explained through the less favorable stress distribution and inferior stress transfer capability over a greater surface area in comparison to the other experimental groups, RB, PV, and CB. The lower strength properties of the flowable composite in comparison to the higher-strength nanohybrid Filtek Z250 composite filler used in the CB group is another factor to consider when comparing the results of the FL and CB groups.

In a different study, Kaur et al. found that a study group in which incisal fragments were reattached with flowable composite exhibited the second-best fracture resistance after the control group of sound teeth. Other test groups included the use of cyanoacrylate adhesive and of cyanoacrylate adhesive in combination with flowable composite in a circumferential chamfer [55]. The current findings that the PV group, with the use of palatal veneers, had comparable and superior FR to the CB and FL groups, respectively, can be explained with regard to the stress distribution and stress transfer performances of the loaded complex in each group. The effectiveness reported by Yikilgan et al. of the palatal veneer technique for reattaching fractured fragments apparently results from their different methodology and study composition [56].

Unlike the FR outcome, the BS of the resin composite buildup group (CB) and sound teeth (NC) was similar, and the 75% prevalence of the mixed mode of failure in resisting the debonding of orthodontic brackets indicates comparable bonding quality in both cases. This is in agreement with previous findings showing that the orthodontic composite bond strength can be improved by using certain composite surface treatments. These treatments include using diamond burs to alter the roughness, as performed in the present study [57,58,59]. Further dissimilar responses between BS and FR were observed: the RB group showed the second-best BS among the experimental groups, with a 50% prevalence of the adhesive mode of failure, followed by the FL and PV (the worst) groups, with a 62% prevalence of the adhesive mode of failure for both. Nevertheless, orthodontic bracket debonding in the CB group was not accompanied by a loss of composite buildup retention in any of the test specimens. In the meantime, one, four, and six specimens exhibited incisal fragment detachment with or without complete bracket separation in the RB, PV, and FL groups, respectively. In both the FR and BS tests, the specific failure pattern in each test group was related to the orientation of the stresses induced in the respective tests, the stress concentration at the interfaces, the crack initiation and propagation within the loaded complex structure, and the ability of the reattachment mechanism in each test group to cross-link the structural discontinuity at the fragment reattachment site [60]. The ultimate strength, resilience, and toughness specific to each component material in the loaded complexes in the FR or BS loading models are decision-making factors [61,62,63]. The nature and direction of the applied forces appeared to have major influence on the integrity of the loaded complex, both in the fracture resistance tests of the reattached incisors and in the orthodontic bracket bond strength tests. This may explain our interesting finding of a negative correlation between the fracture resistance after the reattachment of fractured incisal fragments and the bond strength of orthodontic brackets. These findings could have significant clinical relevance during orthodontic treatment, such as in the activation of orthodontic wires, bracket debonding, and the retention phase. To the best of our knowledge, the correlation between FR and BS has not been previously investigated. Future 3D finite element analysis studies can provide a deeper understanding of the stress distribution and strain and failure criteria for each suggested fragment reattachment technique.

The significant differences among the study groups in both FR and BS, along with the negative correlation between FR and BS, support the rejection of the null hypotheses stating that Ribbond bands have no effect on the FR or BS and that no correlation exists between them. The promising efficacy of using Ribbond bands is offset by the complexity of their application, which requires prior slot preparation, compared to the simpler and more affordable flowable composite application used in the FL group.

Although increasing the sample size reduces variability and assures the reliability and reproducibility of results [64], collecting maxillary central incisors extracted from humans that met the assigned selection criteria of this study was also a challenge. The limitations of the current study include unavoidable variations in teeth size and structure, despite choosing teeth of a similar size and extracted from patients of the same age group; the use of static loading until fracture, which does not simulate the clinical situation; and the exclusion of aging, which was beyond the scope of this study. Future research should consider how aging under oral environmental conditions can affect both the fracture strength of reattached incisal fragments and the bonding effectiveness of orthodontic brackets. In addition to saliva and oral microbial biochemical activities, different loading patterns during mastication, loads, temperatures, and pH cycling, as well as the adoption of oral hygiene measures like tooth brushing, are potential influences [65]. Micromorphological analysis through SEM of failed interfaces could have provided broader information and deeper analysis. In addition, although the immersion of extracted teeth in sodium hypochlorite is an effective disinfection method, it may negatively affect adhesion quality, as sodium hypochlorite is a strong oxidizing agent that disrupts the free radical polymerization process [66].

## 5. Conclusions

Under the circumstances of the present study, and considering all its limitations, the following conclusions can be drawn:

1- Among the incisal fragment reattachment protocols investigated, using Ribbond polyethylene fiber bands seems to provide the best performance in reattached tooth reinforcement and the best orthodontic bracket bond strength.
2- Using resin composite buildup to restore a fractured incisor appears to provide the best bonding quality for orthodontic brackets.
3- Tooth fracture resistance and orthodontic bracket bond strength seem to be inversely proportional to each other.

These implications suggest that dental professionals must carefully select materials and protocols, with consideration of the specific characteristics of each clinical case, in order to ensure effective and long-lasting treatment outcomes.

## Figures and Tables

**Table 1:** Materials used in this study.

Material	Composition	Manufacturer	Lot No.
3M Scotchbond universal etchant, etching gel	35% orthophosphoric acid	3M, Deutschland GmbH, Neuss, Germany	9513767
3M Adper Single Bond 2 Adhesive, single-bond adhesive	BisGMA, HEMA, dimethacrylates, ethanol, water, a novel photoinitiator system, and a methacrylate functional copolymer of polyacrylic and polyitaconic acids	3M ESPE, St. Paul, MN, USA	NF18673
Filtek Z250 Universal Restorative, nanohybrid composite resin	Bis-GMA (Bisphenol A diglycidyl ether dimethacrylate) and a blend of UDMA (urethane dimethacrylate) and Bis-EMA(Bisphenol A polyethylene glycol diether dimethacrylate)	3M ESPE, St. Paul, MN, USA	NF15836
Filtek Supreme flowable composite, flowable composite	Procrylat, BisGMA, and TEGDMA resins. The fillers were a combination of a non-agglomerated/non-aggregated surface-modified 20 nm silica filler, a non-agglomerated/non-aggregated surface-modified 75 nm silica filler, an aggregated surface-modified zirconia/silica cluster filler (comprising 20 nm silica and 4 to 11 nm zirconia particles), and an ytterbium trifluoride filler with particle sizes ranging from 0.1 to 5.0 μm. The aggregate had an average cluster particle size of 0.6 to 10 μm. The total inorganic filler loading was approximately 65% by weight (46% by volume).	3M ESPE, St. Paul, MN, USA	NF20803 10/2024
Ribbond THM, reinforcement ribbon	Ultra-High-Molecular-Weight Polyethylene-Polyethylene, Homopolymer H-(CH2-CH2)n-H	Ribbond, Inc., Seattle, WA, USA	00236334430
PINNACLE™, stainless steel orthodontic brackets	Stainless steel	Ortho Technology Inc, West Columbia, SC, USA	1026793
Orthodontic bracket adhesive and primer system: I-Transbond™ PLUS Colour Change Adhesive II-Transbond™ XT Light Cure Adhesive Primer	I-Bisphenol A Diglycidyl Ether Dimethacrylate (BisGMA), Triethylene Glycol, Dimethacrylate (TEGDMA), Silane-treated quartz, photoinitiators for light curing, and color-changing agents to assist in positioning and excess clean-up	3M Unitek, Monrovia, CA, USA	NF20792
	II-Bisphenol A Diglycidyl Ether Dimethacrylate (BisGMA): 45–55%; Triethylene Glycol Dimethacrylate (TEGDMA): 45–55%; -4-(Dimethylamino)-Benzeneethanol: <0.5%		9178214

**Table 2:** FR means (N) and BS means (MPa) ± standard deviation (SD).

Group	Fracture Resistance Mean (N) ± SD	Significant Differences *	Shear Bond Strength Mean (MPa) ± SD	Significant Differences *
Negative control (sound tooth) (NC)	514.4 ± 19.4	A	17.6 ± 0.6	A
Composite buildup (CB)	224.9 ± 23.8	C	16.6 ± 0.5	B
Flowable composite (FL)	203.7 ± 7.8	D	13.4 ± 0.8	D
Palatal veneer (PV)	234.6 ± 6.6	C	6.5 ± 0.9	E
Ribbond band (RB)	324.6 ± 20.9	B	15.2 ± 0.7	C

* The same letters within one column denote no statistically significant differences between groups.

**Table 3:** Failure mode percentage (%) of total failure in fracture resistance.

Group	Cohesive Failure (%)	Adhesive Failure (%)	Mixed Failure (%)	Substrate (%)
CB	25	12.5	37.5	25
FL	25	25	50	0
PV	12.5	62.5	12.5	12.5
RB	0	25	37.5	37.5

**Table 4:** Failure mode % of total failure in orthodontic bracket bond strength.

Group	Cohesive Failure (%)	Adhesive Failure (%)	Mixed Failure (%)
NC	0	25	75
CB	0	25	75
FL	0	62.5	37.5
PV	0	62.5	37.5
RB	0	50	50

**Table 5:** Frequency of incisal fragment detachment upon orthodontic bracket debonding.

Groups	Bracket Debonding with Incisal Fragment	Bracket Debonding Without Incisal Fragment
Composite buildup (CB)	0	8
Flowable composite (FL)	6	2
Palatal veneer (PV)	4	4
Ribbond band (RB)	1	7

## Data Availability

The data of the research is available upon request from the corresponding author.
